# Benzodiazepine Misuse Among Health Care Workers: The Effect of Sleep Disorders on Work Performance

**DOI:** 10.3390/jcm14124266

**Published:** 2025-06-16

**Authors:** Carlos Roncero, José Lorenzo Bravo-Grande, Pilar Andrés-Olivera, Marta Peña, Carlos Treceño, Pilar González-Pelaez, Lourdes Aguilar, Diego Remón-Gallo, Armando González-Sánchez

**Affiliations:** 1 Department of Health Sciences, Miguel de Cervantes European University (UEMC), Valladolid (Spain), C. del Padre Julio Chevalier, 2, 47012 Valladolid, Spainctreceno@uemc.es (C.T.); mpgonzalez@uemc.es (P.G.-P.); 2 Instituto de Investigación Biomédica de Salamanca (IBSAL), Hospital Virgen de la Vega, 10 ª Planta, Paseo de San Vicente, 58-182, 37007 Salamanca, Spain; jlbravo@saludcastillayleon.es (J.L.B.-G.); mpolivera@saludcastillayleon.es (P.A.-O.); lourdesaguilar@usal.es (L.A.); d.remon@usal.es (D.R.-G.); 3 Psychiatric Unit, School of Medicine, University of Salamanca (Spain), Campus Miguel de Unamuno, Calle Alfonso X El Sabio s/n, 37007 Salamanca, Spain; 4 Network of Research in Primary Care of Addictions (RIAPAD), Instituto Carlos III (Spain), 28029 Madrid, Spain; 5 Department of Occupational Health-Prevention of Occupational Risks, Salamanca Health Area, University of Salamanca Health Care Complex, Paseo de San Vicente 58-182, 37007 Salamanca, Spain; 6 Psychiatry Service, University of Salamanca Health Care Complex, Paseo de San Vicente 58-182, 37007 Salamanca, Spain; 7 Faculty of Psychology, Pontifical University of Salamanca (UPSA), C/Compañía, 5, 37002 Salamanca, Spain

**Keywords:** benzodiazepines, medication use, healthcare workers, insomnia, self-medication

## Abstract

**Background**: Benzodiazepines (BZDs), commonly used to treat insomnia and anxiety, are increasingly used in Spain, raising concerns due to their potential for abuse and dependence. This study investigates the use of BZDs and other psychotropic medications among healthcare workers, exploring their prevalence, associated factors, and their relationship with mental health issues following the COVID-19 pandemic. **Methods**: An anonymous online survey was conducted among healthcare workers at the Salamanca University Healthcare Complex (CAUSA) from March 2023 to January 2024. Of 1121 participants, 685 provided complete responses, which were analysed. Insomnia, anxiety, and depression were assessed using the Insomnia Severity Index (ISI) and Patient Health Questionnaire-4 (PHQ-4). **Results**: Of the respondents, 23.8% reported using sleep medication, with 27.8% doing so without a prescription. Additionally, 14.7% used medication for depression or anxiety, with only 0.6% without a prescription. Hypnotic medicine use was associated with older age, insomnia, anxiety, depression, psychological or psychiatric treatment, COVID-19 after-effects, and diagnosed sleep disorders. Night-shift work was associated with increased hypnotic medication use in men but not in women. The use of these medications was linked to a reduced quality of life and impaired work performance. **Conclusions**: The use of BZD and self-medication are prevalent among healthcare professionals, exceeding the rates observed in the general population. These findings highlight the urgent need for targeted interventions to address psychotropic medication use, promote other pharmacological and non-pharmacological alternatives for insomnia, and enhance mental health support for this vulnerable population.

## 1. Introduction

Benzodiazepines (BZDs) are psychotropic drugs that produce sedative, hypnotic, anxiolytic, antiepileptic, amnestic, and muscle-relaxant effects, and their consumption requires a prescription [1]. They are primarily used in the treatment of anxiety and as hypnotics [2]. The global prevalence of BZD use is approximately 2.2% [3]. Among the Spanish adolescent population, there has been an observed increasing trend in their use, rising from 2.4% to 3.0% between 2004 and 2014 [4]. Moreover, during the COVID-19 pandemic, approximately 40% of the population experienced sleep disturbances and the impact was particularly pronounced among patients with COVID-19, children, and healthcare workers (HCWs) [5]. These sleep difficulties were exacerbated by the COVID-19 lockdown, particularly among HCWs, who experienced severe sleep disturbances, such as poor sleep quality and insomnia [6], especially those directly involved in the care of COVID-19 patients [7].

According to the Organisation for Economic Co-operation and Development (OECD), the defined daily dose per 1000 inhabitants per day (DHD) of hypnotics and sedatives (N05C) in Spain has progressively increased from 26.8 doses in 2010 to 35.2 doses in 2022 [8]. The growing consumption of hypnotics and sedatives (N05C), as well as anxiolytics (N05B), is causing concern [9].

The misuse of these substances is associated with public health problems [10], and overdoses can lead to the progressive impairment of consciousness, slowing down the central nervous system and leading to depression, coma, stupor, nystagmus, apnoea, hallucinations, and hypothermia [11]. Additionally, withdrawal symptoms frequently occur in patients who have used BZDs, even at regular doses, for periods of six months or more [12], but there is currently no widely accepted consensus or standardized guidelines regarding the clinical use of BZDs and opioids [13]. It is estimated that BZD misuse affects approximately 17.2% of users, with consumption without prescription being the most common misuse, often facilitated by a friend or patient [14]. HCWs have easy access to these medications, and this poses significant risks and challenges due to the high levels of dependence observed among HCWs, highlighting the urgent need to study the prevalence of this phenomenon [15]. Those authorized to handle these medications are at increased risk of inappropriate use due to their direct access and frequent exposure [16,17,18,19,20]. In this regard, self-medication is a common practice among hospital personnel, with prevalence rates above 80% among doctors and nurses, and above 70% among administrative staff [21]. Due to the associated risks and their potential for misuse, protocols have been implemented to reduce BZD prescriptions [22].

HCWs are at increased risk of sleep disorders and work-related stress [23]. Many of these individuals who use BZDs may experience difficulties in the future due cognitive impairment even with minimal exposure to BZDs [24]. This may lead to increased benzodiazepine use among nurses, particularly those of older age or with underlying mental health conditions [25].

This study aims to investigate the prevalence of the use of both sleep medications and other psychoactive substances among HCWs, identify the sociodemographic and occupational profiles most associated with their use, and explore the relationship between substance use and sleep problems, depression, and anxiety. It also aims to contextualize findings within the trends of anxiolytic use in Spain. This research is part of a broader project on sleep disorders and their consequences among HCWs [26].

## 2. Materials and Methods

### 2.1. Procedure and Participants

The survey was disseminated via institutional email to all staff members of the University of Salamanca Health Care Complex (CAUSA). Additionally, physical posters (see Supplementary Material S1: “Poster”) were placed within CAUSA facilities, and an announcement was made on the institution’s homepage. The questionnaire remained active, gathering responses from 7 March 2023 to 5 January 2024.

The potential participants (population) included all CAUSA employees. At the time the questionnaire was open, the staff comprised 6193 individuals: 702 medical personnel, 1946 other healthcare professionals, and 3545 non-healthcare workers. Inclusion criteria required participants to be actively employed at the time of completing the questionnaire. Exclusion criteria included participants who had incomplete responses, who completed the questionnaire excessively fast (under four minutes, based on pilot testing), or who had incorrect responses to an attention check item. To establish this time threshold, three of the authors completed the questionnaire as quickly as possible while still reading and answering each item carefully. All three recorded times close to four minutes. Therefore, this duration was proposed as a potential exclusion criterion to identify individuals who might have completed the questionnaire without paying a proper amount of attention. No maximum response time was set, allowing respondents to begin the questionnaire and complete it at a later point. For respondents scoring above eight points (indicative of subclinical insomnia) on the Insomnia Severity Index (ISI), an option was provided to submit their email address and/or telephone number, enabling the Occupational Health Department to contact them directly and offer treatment for sleep-related problems.

### 2.2. Instruments

An online questionnaire was designed, accessible via the intranet and institutional email (see Supplementary Material S2: “CAUSA Sleep Screening Assessment Questionnaire 2022”). The questionnaire was hosted online and could be completed using either a computer or a mobile phone. It included the ISI scale [27] and the Patient Health Questionnaire-4 (PHQ-4) to screen for anxiety and depression [28]. Additionally, sociodemographic questions were included, covering job role, work shifts, consequences of poor sleep quality (if applicable), as well as the use of medications or other substances.

In order for participants to proceed with the questionnaire, they had to accept an informed consent statement presented on the page prior to the start of the questionnaire.

To assess the use of sleep medication, the following item was presented: “I take sleep medication: once a month or more”, with yes/no response options. Those who answered affirmatively were then presented with the following: “The sleep medication I take is prescribed by a doctor”, which also had yes/no options.

An attention check item was included, worded as follows: “This is an attention check question. If you are reading this, please select response 2”.

### 2.3. Statistical Analysis and Sample Size

Preliminary analyses were carried out to determine the type of statistical tests to apply. Specifically, normality was assessed using both the Kolmogorov–Smirnov and Shapiro–Wilk tests simultaneously. In the various analyses, the null hypothesis of normality was not met, preventing the use of parametric tests. Therefore, non-parametric analyses were chosen.

For inferential analyses, the Mann–Whitney U test and the Kruskal–Wallis H test were used to compare differences between independent groups; Spearman’s rho was employed to assess correlations between ordinal variables; and the chi-square test was used to examine independence between categorical variables.

Adjusted standardized residuals were calculated in the chi-square test to identify cells with significant deviations from expected frequencies. These residuals indicate where significant differences exist between observed and expected values under the null hypothesis of independence. The adjusted residuals approximately follow a standard normal distribution (with a mean of 0 and standard deviation of 1), making interpretation straightforward: absolute values greater than 1.96 indicate a significant difference at the 5% level [29]. These values were reported even when the chi-square test itself was not statistically significant. The phi coefficient was also reported for 2 × 2 tables. A significance level of 5% (*p* < 0.05) was set for all analyses, and *p*-values below this threshold were considered statistically significant.

To conduct a more detailed analysis based on age and highlight the contrast between age groups, the sample was divided into three groups according to age quartiles: the first group included participants aged ≤35 years; the second group (including the second and third quartiles) comprised those aged 36 to 53; and the third group consisted of those aged ≥54 years.

The sample size was determined using the following formula:

$$n=\frac{N\cdot Z_{\alpha }^{2}\cdot p\cdot q}{d^{2}\cdot \left(N-1\right)+Z_{\alpha }^{2}\cdot p\cdot q}$$

where:

- *N* is the total population;
- $Z_{\alpha }^{2}$ = 1.96^2^;
- *p* is the proportion of the variable, set at 0.05;
- *q* is the complement of *p*, calculated as *q = 1 – p*;
- *d* is the maximum allowable error (5%).

For the analysis of trends in BZD consumption in the Spanish population, data from the OECD [8] were used, along with Pearson’s r tests and Fisher’s estimation for 95% confidence intervals.

### 2.4. Included Sample

Of the 1121 completed questionnaires, 738 respondents correctly answered the attention check item. Among them, only those participants who completed the entire questionnaire were selected, resulting in a total of 685 subjects. The 4 min criterion was not applied because none of the remaining participants completed the questionnaire in less than that time.

**Figure 1:**
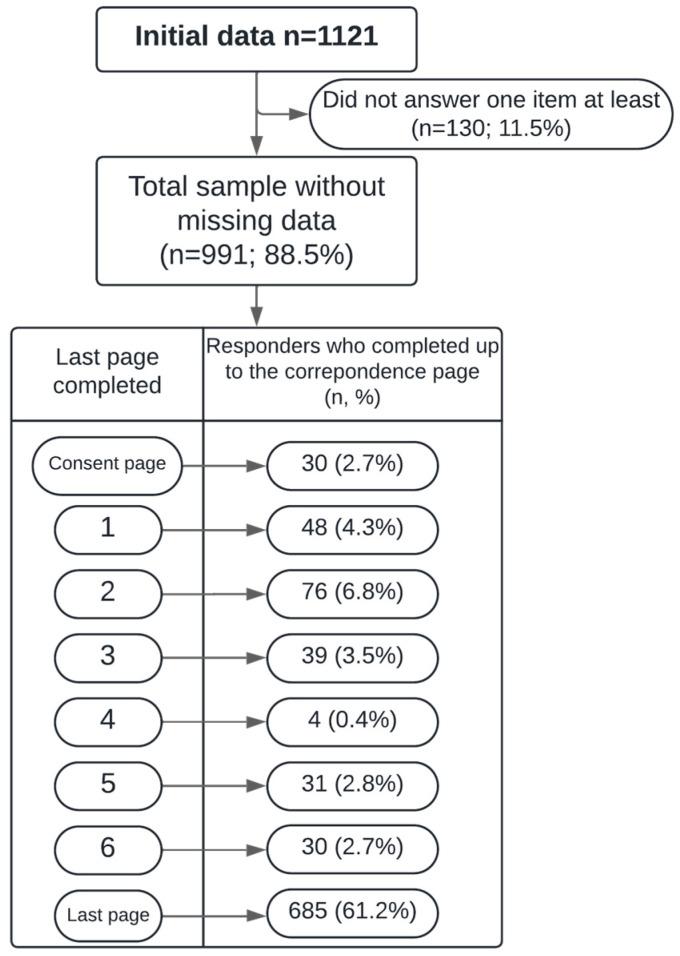
Flow diagram showing the number of respondents based on the last page completed in the questionnaire.

Due to the high number of missing data, an investigation was conducted to determine whether the lack of response was related to any potentially uncomfortable questions in the survey. Of the 7 pages in the questionnaire, 30 respondents did not progress past the informed consent page, 48 did not continue beyond the first page, and the remaining distribution is shown in the figure (Figure 1). No relationships were found between missing data and non-response to questions on the use of illicit drugs. No statistically significant differences were observed between participants’ sex, job position, contract type, or educational level in relation to the last page completed in the questionnaire (see Supplementary Material S3: “Study of ESCAUSA dropout points”).

These 685 responses meet the requirements of having answered all questions, having correctly responded to the attention check item, and having completed the questionnaire in a sufficient amount of time. According to the sample size calculation, a minimum of 362 participants is required for the results to be considered representative; therefore, the findings can be extrapolated to the rest of the population.

The sample consisted predominantly of female workers, aged between 22 and 65 years, with work experience ranging from 0 to 49 years. Most respondents held a university degree and had a permanent employment contract (Table 1).

## 3. Results

### 3.1. Prevalence of Substance Use and Misuse

#### Medication for Treating Sleep Problems

The prevalence of sleep medication use among HCWs was 23.8% (158 individuals): 23.2% among the men and 23.9% among the women.

Among those who take sleep medication, 44 individuals (27.8%) do so without a medical prescription. No significant relationship was found between sex and taking sleep medication at least once a month (whether prescribed or not) (*p* = 0.859).

Additionally, 101 HCWs (14.7%) reported using medication to treat depression and/or anxiety, of whom four individuals (0.6%) used such medication without a prescription.

A significant relationship was found between the presence of sleep disorders and taking sleep medication (*p* < 0.001), as well as having a diagnosed sleep-related illness (*p* = 0.006). However, no significant associations were found between sex and the use of medication either for sleep (*p* = 0.859) or for treating depression or anxiety (*p* = 0.430).

There was a significant association between the use of sleep medication and anxiety disorders (*p* < 0.001), depression (*p* < 0.001), and the use of medication for treating anxiety or depression (*p* < 0.001). Educational level (*p* = 0.147) and job role (such as director, head of a department, and administrative staff member, among others) (*p* = 0.067) did not significantly influence sleep medication use. Similarly, no association was found between the type of job and the use of sleep medication (χ^2^_8_ = 14.63; *p* = 0.067).

No overall differences were observed based on shift work; however, when analysed by sex, differences emerged. Women working rotating (*p* = 0.802) or shift schedules (*p* = 0.479) did not show increased sleep medication use, whereas men working shifts (*p* = 0.007) or night shifts (*p* = 0.023) were more likely to use such medication.

Those who reported using sleep medication once a month or more were older (mean: 47.53; SD: 11.10) than those who did not (mean: 43.88; SD: 11.74) (*p* < 0.001). A more detailed analysis by age group was conducted (Table 2).

Additionally, older workers were more likely to use medication to treat depression, anxiety (*p* = 0.004), or sleep problems (*p* < 0.001). For depression or anxiety, the mean age was 47.84 years (SD = 10.61) among those using medication, compared to 44.19 years (SD = 11.79) among those not using it. For sleep problems, the mean age was 47.53 years (SD = 11.10) for users of sleep medication, compared to 43.88 years (SD = 11.74) for non-users.

### 3.2. Relationship Between Sleep Medication Use, Quality of Life, and Work Impact

It was found that individuals who took sleep medication experienced a greater negative impact on their quality of life, made more errors during daily tasks, felt more overwhelmed by work demands, and had more difficulty performing their job, managing household tasks, and interacting with others. In contrast, sleep medication use was not influenced by being on call, working rotating night shifts, working nights, or high attentional demands.

Additionally, the use of medication for sleep problems was influenced by the presence of sleep disturbances, depression, anxiety, years of experience in the position, and age. However, the duration and frequency of naps, as well as whether or not rest breaks were taken, showed no influence on sleep medication use (see Table 3).

#### 3.2.1. Use of Other Psychotropic Medications

A total of 14.7% of participants reported using medication to treat depression and/or anxiety. No significant associations were found between sex and the use of such medication (*p* = 0.430). Regarding educational level, the profile of workers who use medication for depression or anxiety (χ^2^_5_ = 16.18; *p* = 0.006) includes those with secondary education (adjusted residual = +2.2) or vocational/technical training (adjusted residual = +2.4). In contrast, individuals with a university degree or licentiate are less likely to use psychotropic drugs (adjusted residual = −3.1).

Although the chi-square test did not show a statistically significant association between education level and sleep medication use (χ^2^_5_ = 8.18; *p* = 0.147), the adjusted residuals indicated a higher tendency among those with a technical education (adjusted residual = +2.1) and a lower tendency among professionals in managerial roles (heads of a service or unit) (adjusted residual = −2.4).

Regarding job role and the use of medication for depression or anxiety, no statistically significant relationship was detected (χ^2^_5_ = 12.49; *p* = 0.130). However, an analysis of adjusted residuals showed a higher tendency to consume such medication among administrative assistants or maintenance staff (adjusted residual = +2.7).

No clear profile was identified for the use of medication for depression or anxiety based on the type of employment contract (χ^2^_4_ = 4.45; *p* = 0.348), working overtime (χ^2^_2_ = 0.399; *p* = 0.819), having worked during the pandemic (χ^2^_1_ = 0.036; *p* = 0.348), or sex (χ^2^_1_ = 0.62; *p* = 0.430) (Table 4).

#### 3.2.2. Use of Other Drugs

Among the subgroup of participants who responded to the substance use questions (665 individuals), 13.5% (90 participants) reported smoking more than one cigarette per day. A higher rate of alcohol consumption was also observed among men (*p* < 0.001) and based on age (*p* = 0.006), with younger individuals reporting greater alcohol consumption (see Table 3). Six workers (0.9%) stated that they consumed cannabis once a month or more, and eight (1.2%) reported using illegal depressants (such as opioids, hallucinogens, and similar substances). No participants reported using stimulants (cocaine or amphetamines) or combinations of these substances (see Table 5).

## 4. Discussion

There is a high prevalence of sleep medication use among HCWs (23.8%), with 27.8% of this use occurring without medical supervision—clearly exceeding that observed in the general population and according to its projected trends. Additionally, sleep problems, anxiety, or being under treatment for these conditions, as well as having COVID-19 sequelae or a diagnosed sleep disorder, increase the likelihood of using hypnotic/sedative medication. Among women, hormonal imbalances related to menopause were associated with increased use, while for men, working night shifts or rotating schedules was a contributing factor. Older professionals were more likely to use medication to treat depression, anxiety, and sleep difficulties. The prevalence of tobacco, alcohol, xanines, cannabis, or illegal drugs among healthcare professionals is lower than the general population.

### 4.1. Benzodiacepine Consumption

The high use of benzodiazepines among HCWs aligns with the trends observed in the general Spanish population, in which there is a documented progressive increase in BZD use—a trend also seen in countries such as Finland, Iceland, and Norway [9]. This should be regarded as a public health issue [10].

The high rate of sleep medication use (23.8%) is consistent with previous studies reporting a high prevalence of sleep disorders—especially insomnia—among healthcare professionals, often attributed to night shifts and extended work hours [30,31]. Consequently, this contributes to the elevated use of hypnotic medications. According to the Sustainable Development Goals of the 2030 Agenda, this widespread use should prompt strengthened prevention measures against misuse, ensuring medications are prescribed only when necessary and under medical supervision, providing accessible healthcare services for those who develop dependence (including detox therapies and psychological support), and implementing monitoring systems to prevent misuse and illegal trafficking with the aim of reducing risks associated with these substances [32].

Among hypnotic medicine users, 27.8% reported using sleep medication without a prescription, while only 0.6% did so for depression and/or anxiety. This frequent non-prescription use for sleep suggests a tendency toward self-medication in this group—possibly due to having limited access to or time for regular medical consultations. This phenomenon has been documented in other studies [33], highlighting the need for targeted interventions to improve the sleep quality of night-shift workers.

As expected, sleep problems—especially insomnia—are associated with the greater use of sleep medication among HCWs, in line with previous findings (add references). However, naps and rest breaks did not show a significant effect, suggesting that napping—regardless of its frequency or duration—functions as a compensatory strategy [34].

No significant sex differences were found in the use of benzodiazepines or other psychotropic drugs, although previous studies have reported that women tend to consume more benzodiazepines [35]. This could be explained by biological factors, additional stress faced in work environments [36], hormonal influences and stress perception [37], or a higher prevalence of sleep problems and ADHD among women [38].

No differences were found in sleep medication use based on the contract type or shift work (night shifts or rotating shifts). However, previous research has indicated that shift work, particularly at night, negatively impacts sleep, increases fatigue, and affects the health of healthcare professionals [39]. This type of schedule is associated with insomnia and daytime sleepiness, commonly known as shift work sleep disorder [40,41]. In most studies, women tend to report more sleep problems than men and show a higher risk of depressive symptoms [36,42]. However, our data indicate that only men working night or rotating shifts are more likely to consume benzodiazepines, suggesting that other factors—such as cultural influences or specific work environment conditions—may play a role. This discrepancy could also stem from some workers having developed coping strategies for shift schedules or using non-pharmacological methods to manage sleep. The implementation of forward-rotating shift schedules (day > evening > night > rest) has also been proposed to reduce circadian rhythm disruption and ease their adaptation to different shifts [43]. Further strategies to improve sleep quality in shift workers include maintaining a dark, quiet, and cool sleep environment during the day and using earplugs and eye masks [43].

No increased use was found according to professional category or role, except for lower consumption among senior or managerial staff, who reported less frequently using sleep medication. Previous studies have described nurses as one of the groups most affected, as also noted by Huang et al. [44].

Older workers are more likely to use medication to treat depression, anxiety, and sleep problems. This pattern is consistent with other research [45] that reports increased psychotropic use with age, possibly due to the accumulation of stress, coexisting physical health problems, and changes in sleep patterns.

The use of benzodiazepines is shown to have an influence on the risk of making more mistakes and a poorer job performance—findings that are in line with other studies confirming that work overload [46] and insomnia [47] negatively affect performance by increasing the number of errors committed. However, no greater problems were detected in terms of concentration or the need for high-level cognitive abilities, possibly due to the inherently high cognitive demands of the profession, the seriousness of potential consequences, and the workers’ baseline capacities. Previous studies have already suggested that the impact on work and cognitive abilities may vary [48].

Moreover, the use of sleep medication among workers is associated with a greater perceived negative impact on quality of life, an increase in errors performing daily tasks, relational difficulties, and a sense of being overwhelmed by work demands. These findings are consistent with those of another study [49], which documented that pharmacological treatment for anxiety disorders and sleep problems can have adverse effects on daily functional performance. Such effects include daytime drowsiness and a reduced cognitive capacity, which affect the quality of social and family interactions.

As expected, sleep medication use is also associated with the presence of depression or anxiety, being in psychological or psychiatric treatment, experiencing COVID-19 sequelae, hormonal imbalances due to menopause, and sleep disorders. These findings are consistent with research linking mental health comorbidities and physical problems—such as menopause and the after-effects of COVID-19—with a greater risk of insomnia and the need for sleep medication [50].

### 4.2. Other Psychotropic Drugs

A total of 14.7% of participants reported using medication to treat depression and/or anxiety—a figure notably lower than that of those using treatment for sleep problems. Additionally, self-medication for depression and/or anxiety was far less common (0.6%), which may reflect a greater awareness of the risks associated with self-medicating for mental health disorders, or a perception that such conditions require specialist medical care. This could imply that the treatment—or self-treatment—of sleep problems is not regarded with the same seriousness as anxiety or depression.

Workers with secondary or technical education levels were more likely to use medication for depression or anxiety, aligning with previous studies [51], suggesting an association between lower educational attainment, higher levels of occupational stress, and an increased use of antidepressants and anxiolytics.

The prevalence of tobacco use among healthcare professionals at CAUSA (13.5%) is clearly lower than that of the general Spanish population (19.8%) [52] and similar to figures reported in other samples of Spanish [53] and Brazilian HCWs (15.95%) [54].

Regarding alcohol, the threshold for risky consumption is defined as four standard units (UBEs) per day for men and two for women [55]. Since only seven individuals in our sample consume six or more doses per week, a very small percentage can be considered at risk. The percentage of participants who reported not drinking alcohol at all stands at 43.4%, a figure consistent with other studies on Spanish healthcare professionals (45.6%) [56]. This pattern may reflect sociocultural changes, including a reduction in alcohol consumption in countries such as Spain and Italy [57], a trend expected to continue over time [58].

As for caffeine intake, the European Food Safety Authority (EFSA) sets the risk threshold at 400 mg of xanthines per day, equivalent to roughly five cups of coffee [59]. In our sample, only 5.2% exceeded this amount, which is lower than the rates found in other studies that report a prevalence of risky consumption between 8.8% and 22% [60].

There was a low frequency of recurrent cannabis use at CAUSA (0.9%), in contrast to the Spanish general population (8.6%) [61], the European population (3.9%) [62], or previous studies in medical professionals (8%) [63].

The low prevalence of opioid use (1.5%) also stands in contrast with the 4.0% reported in the Spanish population in the past 30 days [64]. This reduced use of depressants reflects a broader trend observed in many countries towards a decline in illicit opioid consumption, such as heroin, due to ongoing efforts to highlight associated risks and promote prevention, treatment, and education programmes [65,66].

No stimulant use was reported, which may be due to the sociodemographic or occupational characteristics of the study population, who may be less inclined or have less access to such substances compared to more socially accepted drugs like hypnotics or alcohol. Previous research [67] has found stimulant use to be more common in academic or recreational settings among young adults, but much less so among older workers or in professional environments where exposure to such substances is limited [68].

### 4.3. Limitations and Strengths

Our study is subject to several limitations. Firstly, the single-centre design may limit the generalizability of our findings to other institutions, geographical areas, or populations. Moreover, survey-based studies are susceptible to selection bias due to varying levels of motivation among participants to express their views.

A self-selection bias may have been introduced through the recruitment strategy, which relied on email invitations, posters, and a study website. Individuals with a particular interest or personal relevance to the study topic may have been more likely to participate, which could affect the representativeness.

A potential limitation of the study is that data collection spanned a period of ten months, encompassing different seasons and even a change in daylight savings time. As a result, the sample was not obtained at a single point in time, which may have introduced temporal variability in the responses.

Due to the cross-sectional design, causal relationships between shift work, benzodiazepine use, COVID-19 sequelae, and insomnia cannot be established. Similarly, no causal links can be asserted between medication use and occupational or health conditions.

Pre-existing mental health conditions and variability in workload may have acted as confounding factors. Further limitations include the potential for recall bias in self-reported sleep assessments and the lack of longitudinal data to determine causality.

Additionally, as the data were collected through self-reported questionnaires, there is a risk of response bias and underreporting—particularly concerning sensitive topics such as self-medication and substance use. In this regard, caution is advised when interpreting the low prevalence of cannabis and other illicit drug use, as the anonymous nature of the survey does not eliminate the possibility of social desirability bias [69].

Nonetheless, these results, drawn from a large sample representing a significant portion of our centre’s healthcare workforce, contribute valuable insights in a field in which real-world workplace data are limited. They may serve as a foundation for future research on the impact of strategies to manage benzodiazepine misuse on HCW performance and daily functioning.

## 5. Conclusions

The use of benzodiazepines is highly frequent in and relevant to healthcare professionals and is closely associated with the presence of insomnia. Their consumption surpasses that of the general population, despite an already increasing trend in recent years.

A high prevalence of self-medication and the uncontrolled use of benzodiazepines has been observed, as well as a link between their use and problems with job performance. Given that the use of sleep medication is associated with symptoms of anxiety, depression, post-COVID-19 sequelae, menopause, and ongoing mental health treatment, it is essential to adopt a comprehensive approach that addresses both the physical and mental health of HCWs.

In this healthcare context, raising awareness about the inappropriate use of these drugs is vital—particularly among men working shifts and older professionals. To improve sleep and minimize the risk of BZD use, other hypnotic drugs could be used, and individual non-pharmacological strategies are recommended, such as ensuring the possibility of daytime sleep in quiet environments. In line with previous studies [34], healthcare institutions should also actively promote mental health and help regulate the sleep/wake cycle of their staff by introducing measures such as adequate workplace lighting and the encouragement of regular physical exercise.

Finally, specific programmes should be implemented to support professionals with sleep disorders or psychotropic drug use—particularly benzodiazepines—including prevention policies, medical supervision, and strategies to ensure their safe use and professionals’ long-term wellbeing [70].

## Figures and Tables

**Table 1:** Sociodemographic characteristics of the sample, frequencies, and percentages.

Variable	Values
Full sample	(n = 685)
Age (mean, SD)	44.749 (11.70) Range 22–65
Years of experience (average, DT)	12.58 (10.96) Range 0–49
Sex (n, %)	
Man	146 (21.3)
Woman	539 (78.7)
Education level (n, %)	
Primary	19 (2.8)
High school	34 (5.0)
Technical studies	137 (20.0)
University Bachelor’s Degree	340 (49.6)
Master’s	100 (14.6)
Doctorate	55 (8.2)
Type of contract (n, %)	
Owner	299 (43.6)
Indefinite (interim-long-term position, secondment, etc.)	219 (31.9)
Temporary	122 (17.9)
Intern Resident (MIR/PIR/FIR/QIR/BIR/EIR)	37 (5.4)
Other/Unknown	8 (1.2)

Notes: MIR: Medical intern resident; PIR: Psychology intern resident; FIR: Pharmacy intern resident; QIR: Chemistry intern resident; BIR: Biology intern resident; EIR: Nursing intern resident. All of the intern residents were professionals in training.

**Table 2:** Use of sleep medication according to sociodemographic variables and work habits by age group and sex. *p*-values from within-group analyses.

Variables		Age		Sex		Total
	≤35	36 to 53	≥54	Male	Female	
Sex	0.075	0.872	0.054	-	-	0.859
Gender	0.189	0.872	0.054	-	-	0.841
Level of education	0.233	0.391	0.388	0.414	0.010 *	0.147
Job role	0.666	0.259	0.283	0.198	0.069	0.067
Working night shifts	0.894	0.385	0.259	0.023 *	0.479	0.697
Being on call	0.190	0.357	0.546	0.177	0.426	0.093
Current psychiatric treatment	<0.001 **	<0.001 **	<0.001 **	0.035 *	<0.001 **	<0.001 **
Ongoing health issues because of COVID-19	0.013 *	0.059	0.029 *	0.596	<0.001 **	<0.001 **
Diagnosed with a sleep disorder	0.450	0.679	0.005 **	0.194	0.012 *	0.006 **
Missed work due to poor sleep	0.076	0.062	-	0.188	0.071	0.030 *
Frequently changing work shifts that affect sleep	0.610	0.114	0.251	0.007 **	0.802	0.336
Menopausal hormonal imbalance	0.682	0.039 *	0.631	-	0.002 **	0.002 **
Take naps	0.241	0.220	0.035	0.372	0.009 **	0.006 **
Weekly frequency of naps	0.479	0.690	0.737	0.555	0.939	0.773
Average nap duration	0.007 **	0.690	0.737	0.765	0.014 *	0.108
Regular use of medication for depression or anxiety	<0.001 **	<0.001 **	<0.001 **	< 0.001 **	<0.001 **	<0.001 **
Medication for depression or anxiety is prescribed	0.488	0.060	0.573	0.412	0.160	0.422
Regular use of illegal stimulants	-	-	-	-	-	-
Regular use of illegal depressants	0.234	0.008 **	0.782	-	0.081	0.079
Regular use of cannabis-type substances	0.003 **	0.008 **	-	0.071	0.002 **	<0.001 **

Note: *p*-values are due to the inability to perform the analysis or insufficient sample size. * Significance level below 0.05. ** Significance level below 0.01.

**Table 3:** Differences between those who take sleep medication once a month or more and those who do not in terms of means and standard deviations.

Dimension	Variable	*p*-Value	Takes Medication	Does Not Take
Work Demands	Sleeping problem affects quality of life	<0.001 **	7.15 (2.72)	4.74 (3.45)
	Makes more mistakes during daily tasks	<0.001 **	4.40 (3.00)	2.78 (2.80)
	Has trouble focusing at work	<0.001 **	4.33 (3.30)	2.71 (2.80)
	Difficulty performing work	<0.001 **	0.86 (0.71)	0.43 (0.56)
	Has trouble doing chores at home	<0.001 **	1.13 (0.88)	0.64 (0.75)
	Difficulty with social interaction	<0.001 **	1.15 (0.87)	0.55 (0.69)
	Needs high levels of concentration	0.755	8.07 (2.27)	7.80 (2.61)
	Experiences serious consequences due lack of attention	0.768	7.0 (3.23)	6.52 (3.44)
Sociodemographic Characteristics and Sleep Hygiene	ISI Score	<0.001 **	15.52 (4.98)	9.60 (5.52)
	Depression	<0.001 **	2.73 (1.75)	1.63 (1.52)
	Anxiety	<0.001 **	3.06 (1.83)	1.91 (1.65)
	Years of experience in the position	0.004 **	13.93 (11.36)	11.99 (10.81)
	Age	0.006 **	47.13 (11.08)	43.80 (11.75)
	Nap duration	0.108	2.98 (1.57)	3.21 (1.27)
	Taking frequent breaks at work	0.354	3.21 (2.77)	3.70 (2.95)
	Nap frequency	0.773	4.61 (1.935)	4.45 (1.964)

** Significance level below 0.01.

**Table 4:** Adjusted residuals from contingency tables for individuals taking medication to treat depression or anxiety or to aid sleep.

Variable Type	Response Options	Takes Medication for	
		Depression/Anxiety	Sleep
Education	Primary education	−0.6	−0.3
	Secondary education	2.2 *	1.1
	Technical training	2.4 *	2.1 *
	Bachelor’s degree	−3.1 **	−1.4
	Master’s degree	1.3	0.2
	Doctorate	−0.8	−1.5
Job Role	Executive staff	−1.0	−1.4
	Heads of service or unit	0.1	−2.4
	Other C1 and C2 staff	−1.7	−1.0
	Specialist graduates	0.2	1.4
	A2. Nurses, occupational therapists, nutrition graduates, speech therapists	−1.1	−1.3
	C1 and C2. Senior technician, auxiliary nursing technician, pharmacy technician	0.3	1.3
	Management and service staff A1, A2, C1, C2, and OAP	−1.0	−0.8
	Administrative assistants, maintenance staff	2.7 **	1.5
	Other/Unknown	−0.8	−0.2
Type of Employment Contract	Permanent contract	0.4	1.7
	Open-ended (long-term temporary/post-holder replacement)	0.7	−1.2
	Temporary contract	−0.1	−0.8
	Resident (MIR/PIR/FIR/QIR/BIR/EIR)	−1.7	0.6
	Other/ Unknown	−1.2	−0.8
Worked Overtime	Yes, and they are paid	0.3	0.4
	Yes, but they are unpaid	0.5	1.2
	No	−0.6	−1.4
Worked at CAUSA During the COVID-19 Pandemic	Yes	0.2	0.0
	No	−0.2	0.0

Notes: Based on the standard normal distribution, absolute standardized test statistics exceeding ±1.96 correspond to *p*-values below 0.05, indicating statistically significant differences at the 5% level. A1/A2: University graduates (Bachelor’s degree or diploma); C1/C2: Technicians or administrative staff by education level; OAP: Other auxiliary personnel (maintenance, cleaning, etc.). MIR: Medical intern resident; PIR: Psychology intern resident; FIR: Pharmacy intern resident; QIR: Chemistry intern resident; BIR: Biology intern resident; EIR: Nursing intern resident. All of the intern residents were professionals in training. CAUSA: University of Salamanca Health Care Complex. * Significance level below 0.05. ** Significance level below 0.01.

**Table 5:** Weekly alcohol consumption and daily xanthine intake by sex and age.

Substance	Consumption Frequency or Dose	Sex				Age	
		Men	Women	Total	*p*-Value	Mean (SD)	*p*-Value
Alcohol (weekly doses)	Does not drink at all	34 (5.1)	263 (39.5)	297 (43.4)	<0.001 **	44.98 (11.59)	0.010 *
	Drinks 3 or fewer doses	69 (10.4)	226 (34.0)	295 (43,1)		43.70 (11.63)	
	Drinks between 3 and 6 doses	32 (4.8)	34 (5.1)	66 (9,6)		48.91 (11.60)	
	Drinks more than 6 doses	3 (0.5)	4 (0.6)	7 (1,0)		39.43 (11.14)	
	Total	138 (20.8)	527 (79.2)	665 (100)		44.75 (11.70)	
Xanthines (daily doses)	0	25 (3.8)	78 (11.7)	103 (15.5)	0.486	43.34 (11.18)	0.799
	1	38 (5.7)	142 (21.4)	180 (27.1)		44.76 (12.35)	
	2	28 (4.2)	182 (27.4)	210 (31.6)		46.90 (10.93)	
	3	20 (3.0)	74 (11.1)	94 (14.1)		43.64 (12.48)	
	4	14 (2.1)	30 (4.5)	44 (6.6)		41.02 (10.98)	
	5	6 (0.9)	12 (1.8)	18 (2.7)		42.06 (11.46)	
	6	5 (0.8)	4 (0.6)	9 (1.4)		47.89 (12.04)	
	More than 6	2 (0.3)	5 (0.8)	7 (1.1)		41.57 (9.07)	

* Significance level below 0.05. ** Significance level below 0.01

## Data Availability

The data presented in this study are available upon request from the corresponding author. The data are not publicly available due to privacy or ethical restrictions.

## Supplementary Materials

The following supporting information can be downloaded at: https://www.mdpi.com/article/10.3390/jcm14124266/s1. Supplementary S1: poster that was put on the hospital walls; Supplementary S2: Sleep Screening Assessment Questionnaire; Supplementary S3: Study of ESCAUSA dropout points.

## Abbreviations

The following abbreviations are used in this manuscript:

BZD	Benzodiazepines
OECD	Organisation for Economic Co-operation and Development
DHD	Daily dose per 1000 inhabitants per day
N05C	Hypnotics and sedatives
N05B	Anxiolytics
HCW	Healthcare workers
CAUSA	Salamanca University Healthcare Complex
ISI	Insomnia Severity Index
PHQ-4	Patient Health Questionnaire-4
A1/A2	University graduates (Bachelor’s or diploma)
C1/C2	Technicians or administrative staff by education level
OAP	Other auxiliary personnel (maintenance, cleaning, etc.)
MIR	Medical intern resident
PIR	Psychology intern resident
FIR	Pharmacy intern resident
QIR	Chemistry intern resident
BIR	Biology intern resident
EIR	Nursing intern resident
