# Supplementary material for: Benzodiazepine Misuse Among Health Care Workers: The Effect of Sleep Disorders on Work Performance
Source: J Clin Med. 2025 Jun 16;14(12):4266. doi: 10.3390/jcm14124266 (PMC12194565; doi:10.3390/jcm14124266)
Supplement: Supplementary file 1 [file jcm-14-04266-s001.zip › jcm-3669228-supplementary.pdf]

Universidad Europea Miguel de Cervantes

Supplementary files

Poster

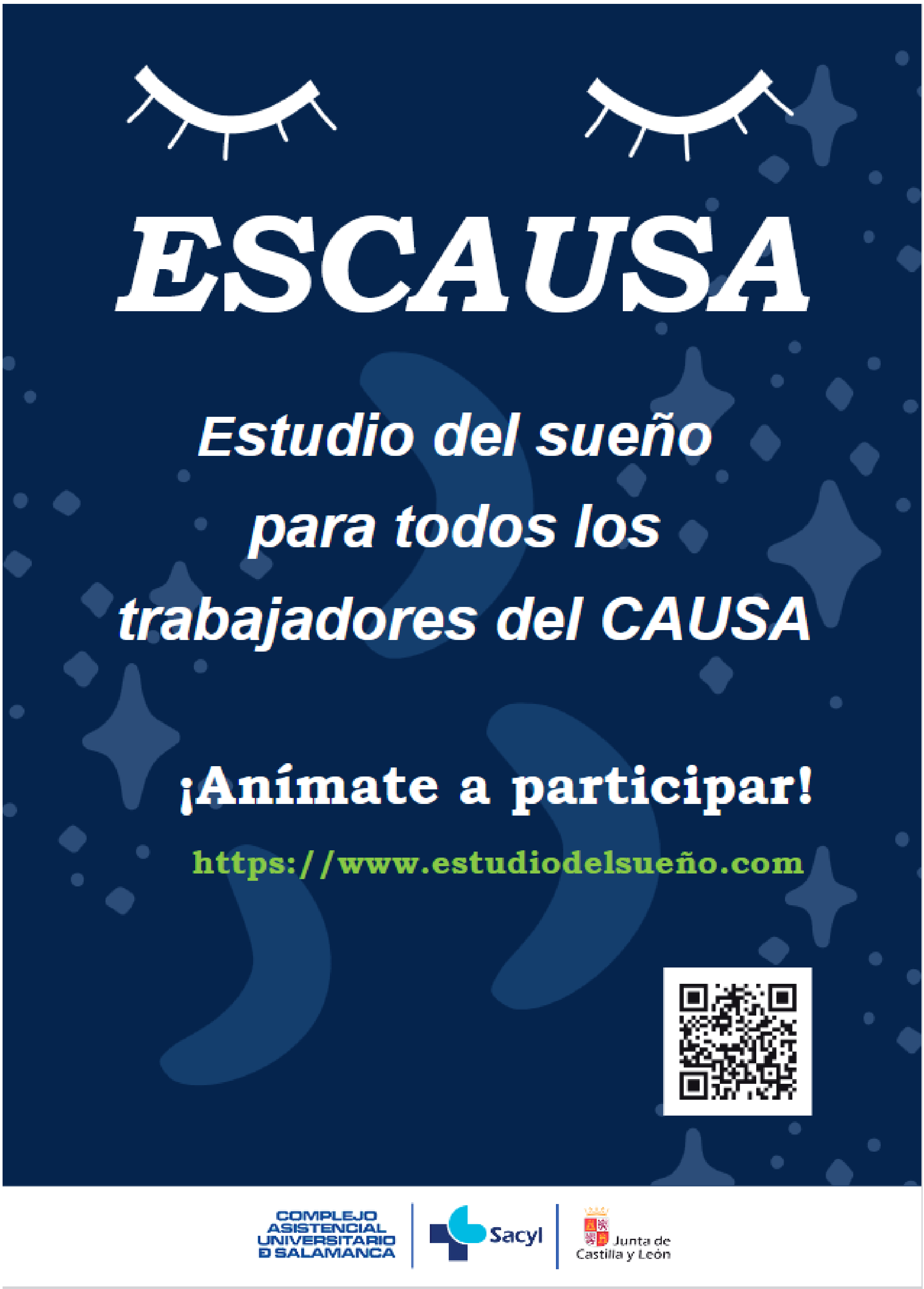

CAUSA Sleep Screening Assessment Questionnaire 2022

Note: The answer options that have the ends of 0 and 10 indicated refer to the fact that it is possible to select the intermediate options.

Sociodemographic

number	Instructions	Item Wording 1	Item 2 Wording	Answer Options
1			Indicate your age in years	[years]
2	This item conditions the i51 *I have hormonal disorders related to menopause.*		What is your biological sex?	(1) Male;<br>(2) Women
3			What gender do you identify with?	(1) I feel like a man;<br>(2) I feel like a woman;<br>(3) Other
4			What is your completed level of studies?	1. Primary<br>2. High school<br>3. Technical studies,<br>4. University degree/bachelor's degree,<br>5. Master<br>6. Doctorate
6		Putting together all the time he worked for CAUSA (even having discontinued contracts)	Please indicate your years of experience working at the CAUSA. In the case where you have worked for less than a year, mark ZERO.	[years]
7		Regarding the current contract	What type of position are you filling?	1. A1 and A2 management staff (directors, deputy directors)<br>2. Heads of service and unit A1 and A2 (Heads of Service, Section and Unit, supervisors, post complement).<br>3. Others C1 and C2 (group, team and personnel leaders, maintenance manager, technician coordinator, installation driver).<br>4. A1 health personnel (Specialist graduates).<br>5. A2 health personnel (nurses, occupational therapists, graduates in nutrition, speech therapists).<br>6. C1 and C2 health personnel (Senior Technician, Auxiliary Care Technician, Pharmacy Technician).<br>7. Management and service staff A1, A2, C1, C2 and OAP (Graduates, Engineers and Librarians; Technical Engineers. Industrial or similar degree).<br>8. Administrative Assistant, maintenance personnel, Porters, Operators, Assistants).
8		Regarding the current contract	Indicate the type of contract you have	1. Owner<br>2. Indefinite (interim-long-term position, secondment, etc.)<br>3. Temporary<br>4. Residente (MIR/PIR/FIR/QIR/BIR/EIR).
9		Regarding the current contract	In the last month, have you worked overtime?	(1) Yes;<br>(2) No
10		Regarding the current contract	In the last month, have you worked more hours than you are contractually entitled to?	(1) Yes;<br>(2) No
11	This item conditions the i68 *Have you been worried because your job was in danger due to the COVID-19 pandemic?*		Have you worked at CAUSA during the first waves of the COVID-19 pandemic?	(1) Yes;<br>(2) No

drcarlosroncero@gmail.com

ISI Insomnia Severity Index

https://doi.org/10.1016/j.sleep.2011.06.019

The sum of responses to ISI items (from 12 to 18) conditions item 70.

number	Item Wording 1	Item 2 Wording	Answer Options
12	Please indicate the SEVERITY of your current (e.g., within the last 2 weeks) sleep problem(s):	Difficulty falling asleep	(0) Nothing;<br>(1) Mild;<br>(2) Moderate;<br>(3) Severe;<br>(4) Very serious
13	Please indicate the SEVERITY of your current (e.g., within the last 2 weeks) sleep problem(s):	Difficulty staying asleep:	(0) Nothing;<br>(1) Mild;<br>(2) Moderate;<br>(3) Severe;<br>(4) Very serious
14	Please indicate the SEVERITY of your current (e.g., within the last 2 weeks) sleep problem(s):	Waking up too early	(0) Nothing;<br>(1) Mild;<br>(2) Moderate;<br>(3) Severe;<br>(4) Very serious
15		How **SATISFIED** are you with your dream today?	(0) Very satisfied;<br>(1) Satisfied;<br>(2) Neutral;<br>(3) Not very satisfied;<br>(4) Very dissatisfied
16		To what extent do you feel your sleep problem **INTERFERES** with your daily functioning (e.g., daytime fatigue, ability to perform daily tasks/work, concentration, memory, mood, etc.)?	(0) Nothing;<br>(1) A little;<br>(2) Something;<br>(3) A lot;<br>(4) A lot
17		To what extent do you think **OTHERS are aware** of your sleep problem because of what it affects your quality of life?	(0) Nothing;<br>(1) A little;<br>(2) Something;<br>(3) A lot;<br>(4) A lot
18		How **CONCERNED** are you about your current sleep problem?	(0) Nothing;<br>(1) A little;<br>(2) Something;<br>(3) A lot;<br>(4) A lot

drcarlosroncero@gmail.com

Insomnia Daytime Symptoms and Impacts Questionnaire (IDSIQ)

https://doi.org/10.1007/s40271-020-00474-z

number	Item Writing	Answer Options
19	To what extent have you felt **clear** today?	(0) Nothing clear<br>(10) Very clear
20	To what extent have you felt **worried** today?	(0) Not at all concerned<br>(10) Very concerned
21	How frustrated **have you been** by the lack of sleep today?	(0) Not at all frustrated<br>(10) Not at all frustrated
22	To what extent have you felt **irritable** today?	(0) Not irritable at all<br>(10) Very irritable
23	To what extent have you felt **stressed** today?	(0) Not stressed at all<br>(10) Very stressed
24	To what extent have you felt **energetic** today?	(0) Not at all energetic<br>(10) Very energetic
25	To what extent have you felt **sleepy** today?	(0) Not at all drowsy<br>(10) Very sleepy
26	To what extent have you felt **awake** today?	(0) Not awake at all<br>(10) Very awake
27	To what extent have you felt with renewed **strength** today?	(0) With the strength not at all renewed<br>(10) With very renewed strength
28	To what extent have you felt **physical fatigue** today?	(0) No physical fatigue<br>(10) A lot of physical fatigue
29	To what extent have you felt **mental fatigue** today?	(0) No mental fatigue<br>(10) A lot of mental fatigue
30	To what extent have you been **able to concentrate** today?	(0) I have been completely unable to concentrate<br>(10) I was able to concentrate very well
31	To what extent have you been **forgetful** today?	(0) Not forgetful at all<br>(10) Very forgetful
32	How much has **it cost you to do your daily activities** today (e.g., reading, cleaning, working, studying)?	(0) It didn't cost me anything<br>(10) It has cost me a lot

drcarlosroncero@gmail.com

PHQ2, GAD2 (the union of the two results in PHQ-4), item 15 of PHQ15

- PHQ2 Kroenke, K., Spitzer, R., & Williams, J. (2003). The patient health questionnaire-2 of a two-item screener validity depression. Med Care, 41, 1284–1292.
- PHQ4 Kroenke, K., Spitzer, R. L., Williams, J. B. W., & Löwe, B. (2009). An ultra-brief screening scale for anxiety and depression: The PHQ-4. Psychosomatics, 50(6), 613–621. https://doi.org/10.1176/appi.psy.50.6.613
- GAD2 Williams, J. B. W., Monahan, P. O., Löwe, B., Kroenke, K., Spitzer, R. L., & Lö, B. (2007). Metabolic syndrome View project Frontiers in Psychiatry Research Topic on coronavirus and mental health View project Anxiety Disorders in Primary Care: Prevalence, Impairment, Comorbidity, and Detection. https://doi.org/10.7326/003-4819-146-5-200703060-00004
- PHQ15 Actas Esp Psiquiatr 2010; 38(6):345-57 https://www.researchgate.net/publication/49712122_Validation_of_the_Spanish_Version_of_the_PHQ-15_Questionnaire_for_the_evaluation_of_physical_symptoms_in_patients_with_depression_andor_anxiety_disorders_DEPRE-SOMA_study

number	Item Wording 1	Item 2 Wording	Answer Options
33	Over the past two weeks, how often have you felt discomfort from the following issues?	Little interest or pleasure in doing things.	(0) Not at all;<br>(1) Several days;<br>(2) More than half of the days;<br>(3) Almost every day
34	Over the past two weeks, how often have you felt discomfort from the following issues?	Feeling discouraged, depressed, or hopeless.	(0) Not at all;<br>(1) Several days;<br>(2) More than half of the days;<br>(3) Almost every day
35	Over the past two weeks, how often have you felt discomfort from the following issues?	Feeling nervous, anxious, or very tense.	(0) Not at all;<br>(1) Several days;<br>(2) More than half of the days;<br>(3) Almost every day
36	Over the past two weeks, how often have you felt discomfort from the following issues?	Not being able to stop worrying or not being able to control worry.	(0) Not at all;<br>(1) Several days;<br>(2) More than half of the days;<br>(3) Almost every day
37		Over the past 4 weeks, how much has it bothered you to have trouble sleeping?	(0) Not at all;<br>(1) Several days;<br>(2) More than half of the days;<br>(3) Almost every day

drcarlosroncero@gmail.com

Substances

number	Item Instructions	Item Wording 1	Item 2 Wording	Answer Options 1
38		Smoking regularly	More than 1 cigarette a day	(1) Yes<br>(2) No
39		Weekly I take	Alcohol	1.- No<br>2.- I take 3 or less<br>3.- Volume between 3 and 6<br>4.- I take more than 6
40		The number of coffees/teas/energy drinks I usually drink each day is: (If it's double coffee, count by 2. If it is an energy drink [330ml] count by 5).		(1) 0<br>(2) 1<br>(3) 2<br>(4) 3<br>(5) 4<br>(6) 5<br>(7) 6<br>(8) More than 6
41	This item conditions the i42 *The medication I take to sleep has a medical prescription*	I take 1 time a month or more	Sleep medication	(1) Yes<br>(2) No
42	Only if you take sleep medication (if i41=1)	The medication I take to sleep is prescribed by a doctor		(1) Yes<br>(2) No
43	This item conditions the i44 *The medication I take for depression or anxiety has a medical prescription*	I take 1 time a month or more	Medication for depression or anxiety	(1) Yes<br>(2) No
44	Only if you take medication for depression or anxiety (if i43=1)	The medication I take for depression or anxiety is prescribed by a doctor		(1) Yes<br>(2) No
45		I take 1 time a month or more	Illegal substances STIMULANTS (cocaine, amphetamines...)	(1) Yes<br>(2) No
46		I take 1 time a month or more	Illegal DEPRESSANT substances (opiates or others).	(1) Yes<br>(2) No
47		I take 1 time a month or more	Illegal substances CANNABIS TYPE.	(1) Yes<br>(2) No

drcarlosroncero@gmail.com

Protective or risk factors, quality of life and occupational consequences

number	Item Instructions	Item Wording 1	Item 2 Wording	Answer Options
48		I am currently undergoing psychological or psychiatric treatment		(1) Yes;<br>(2) No
49		I currently have sequelae from having had COVID disease		(1) Yes;<br>(2) No
50		I have been diagnosed with a sleeping sickness		(1) Yes;<br>(2) No
51	Only if sex = female (if i2 = 2)	I have hormonal disorders related to menopause.		(1) Yes;<br>(2) No;<br>(3) Not applicable
52		I currently work night shifts.		(1) Yes;<br>(2) No
53		I do guard duty		(1) Yes. I do physical presence shifts, but not localized;<br>(2) Yes, I do localized guards, but with a non-physical presence;<br>(3) Yes, I do both physical and localized on-call duty.<br>(4) I don't do guard duty.
54		I have rotating shifts that cause me frequent changes in the hours I spend sleeping.		(1) Yes;<br>(2) No
55	This item conditions the i57 and i58	Duermo la siesta.		(1) Yes;<br>(2) No
56	Only you duerme the siesta (si i56=1)	The frequency with which I take a nap in a week is:		1. 0<br>2. 1<br>3. 2<br>4. 3<br>5. 4<br>6. 5<br>7. 6<br>8. 7
57	Only you duerme the siesta (si i56=1)	How long does he take a nap on average?		(1) Less than 15 minutes;<br>(2) Between 15 and 30 minutes;<br>(3) Between 30 and 45 minutes;<br>(4) Between 45 and 60 minutes;<br>(5) Between one hour and one and a half hours;<br>(6) Between an hour and a half and two hours;<br>(7) More than 2 hours
58		In the last month and due to fatigue I have noticed that I make more mistakes in my daily tasks.		(0) Strongly agree;<br>(10) Strongly disagree
59		To perform my work I need high concentration and the use of higher capacities (attention, perception and memory).		(0) Strongly agree;<br>(10) Strongly disagree
60		In the course of my work, there are days of concentrated and sustained attention for long periods of time		(0) Strongly agree;<br>(10) Strongly disagree
61		I take enough breaks during my workday to break the monotony and disconnect to resume proper attention in my work		(0) Strongly agree;<br>(10) Strongly disagree
62		The lack of attention in my work has a direct consequence of serious consequences		(0) Strongly agree;<br>(10) Strongly disagree
**number**	**Item Instructions**	**Item Wording 1**	**Item 2 Wording**	**Answer Options**
63		Sleep negatively influences my quality of life		(0) Strongly agree;<br>(10) Strongly disagree
64		Due to poor sleep quality in the last month	I've missed work	(1) Yes;<br>(2) No

drcarlosroncero@gmail.com

65		In the last month and due to the poor quality of sleep I have difficulty	Doing my job	(0) No difficulty;<br>(1) A little difficulty;<br>(2) A lot of difficulty;<br>(3) Extreme difficulty
66		In the last month and due to the poor quality of sleep I have difficulty	Perform household chores	(0) No difficulty;<br>(1) A little difficulty;<br>(2) A lot of difficulty;<br>(3) Extreme difficulty
67		In the last month and due to the poor quality of sleep I have difficulty	Relate to others	(0) No difficulty;<br>(1) A little difficulty;<br>(2) A lot of difficulty;<br>(3) Extreme difficulty
68	Only if you worked during first waves (if i11=1)	Have you been worried that your job was in jeopardy due to the COVID-19 pandemic?		(0) No worry;<br>(1) A little worry;<br>(2) A lot of concern;<br>(3) Extreme concern
69		In the last month and due to the poor quality of sleep I have felt that the attention that work demands of me is greater than what I can offer.		(0) Strongly agree;<br>(10) Strongly disagree.

drcarlosroncero@gmail.com

Treatment, subsequent contact and final message

number	Item Instructions	Item Writing	Answer Options
70	This item is conditioned by i12 to i18.<br>It will only be shown to those who have obtained a clinical insomnia score (scores greater than 15).<br>*Only if ISI≥15 (sum of ISI test item scores, which are 12 to 18)*<br>Total score categories:<br>0–7 = No clinically significant insomnia<br>8–14 = Subthreshold insomnia<br>15–21 = Clinical insomnia (moderate severity)<br>22–28 = Clinical insomnia (severe)	You are told that you may have problems with sleep.<br>Occupational Medicine has set up an operation to treat specific sleep problems. If you want to be treated for your sleep problem , **please provide your phone number and email.**	[OPEN RESPONSE]

Final message for all participants

Thank you very much for your participation. Below you can find information about sleep hygiene: https://www.cop.es/colegiados/PV00520/Como%20dormir%20mejor.pdf

drcarlosroncero@gmail.com

Study of ESCAUSA dropout points

Below is the table of respondents who stay on each of the pages (table 1).

Table S1. Number of respondents according to their last page answered.

Last answered page	Frequency	Percentage	Valid Percentage	Acumulative Percentaje
0	30	2.7	3	3
1	48	4.3	4.8	7.9
2	76	6.8	7.7	15.5
3	39	3.5	3.9	19.5
4	4	0.4	0.4	19.9
5	31	2.8	3.1	23
6	78	7	7.9	30.8
7	686	61.2	69.2	100
Total	992	88.5	100	
Lost	129	11.5		
Total (including lost)	1121	100		

There are 7 pages in total, of which, 30 respondents did not go beyond informed consent, 48 respondents did not go beyond the first page and so on with the rest as indicated in the table in the frequency column. In the lost section, there are 129 subjects who accessed and did not accept informed consent, among them, only the IP and the date of access were registered.

As can be seen, on the first page, 30 people gave consent, but did not continue to respond (of which 1 responded to sex and gender, but nothing else).

Between those lost (129) and those who only answered the first page (30), there are 159 respondents. Of these, 151 respondents did not respond to the sociodemographic section and 8 responded only to sociodemographic questions.

All this leaves a sample size of 686 subjects, but one was eliminated from this analysis because it was a test answer, leaving 685 subjects.

For the simulation of the questionnaire, members of the research team were evaluated in different pilot tests. The objective was to detect typographical errors, formatting errors and inconsistencies of any kind. In the same way, three response time trials were made, to determine the minimum time required to complete the questionnaire, obtaining a time of 4 minutes. Although this time is mentioned as a filter, no questionnaire was actually removed using this filter because it was the last filter to be applied and no questionnaire was removed using this method.

On the other hand, there are some questions such as those about working shifts that are conditional answer options (they are only presented to those who work in shifts) and that is why they have many absentees (for example, the medication I take has a medical prescription, they are only presented to those who take medication).

Mann-Whitney and Kruskall-Wallis tests was run and did not found differences in the last page answered according to sex (p=0,884), level of insomnia (p=0,396), job role (p=0,051) or type of contract (p=0,869).

In the other hand we found light differences in the last page answered according to educational level (p=0,048). However, these observed differences did not reach statistical significance after applying the Bonferroni correction.

drcarlosroncero@gmail.com

Table S2. Pairwise Comparisons of: What is your highest completed level of education

Sample 1 - Sample 2	Test Statistic	Standard Error	Standardized Test Statistic	Sig.	Adj. Sig.[a]
Primary - Technical studies	-32.213	50.542	-0.637	0.524	1.000
Primary - Secondary	-46.219	57.933	-0.798	0.425	1.000
Primary - Bachelor's degree	-56.407	49.064	-1.150	0.250	1.000
Primary - Doctorate	-88.896	54.026	-1.645	0.100	1.000
Primary - Master's degree	-102.264	51.605	-1.982	0.048	0.713
Technical studies - Secondary	14.006	36.166	0.387	0.699	1.000
Technical studies - Bachelor's degree	-24.194	18.949	-1.277	0.202	1.000
Technical studies - Doctorate	-56.684	29.506	-1.921	0.055	0.821
Technical studies - Master's degree	-70.052	24.795	-2.825	0.005	0.071
Secondary - Bachelor's degree	-10.188	34.069	-0.299	0.765	1.000
Secondary - Doctorate	-42.678	40.893	-1.044	0.297	1.000
Secondary - Master's degree	-56.046	37.636	-1.489	0.136	1.000
Bachelor's degree - Doctorate	-32.489	26.895	-1.208	0.227	1.000
Bachelor's degree - Master's degree	-45.857	21.623	-2.121	0.034	0.509
Doctorate - Master's degree	13.368	31.290	0.427	0.669	1.000

Each row tests the null hypothesis that the distributions of Sample 1 and Sample 2 are equal. Asymptotic significances (two-sided tests) are displayed. The significance level is 0.050.
[a] Significance values have been adjusted for multiple comparisons using the Bonferroni correction.

The least answered striking question is the fear of losing one's job due to COVID, but one possible explanation is that it was the last question.

Table S3. Questions and number of non-respondents

Question number	Question Statement	Lost data
1	If you work shifts, how many hours do you work in your regular shift? [7 Morning, 7 Afternoon and 10 Night]	421
2	If you work shifts, how many hours do you work in your regular shift? [12-hour shifts]	916
3	If you work shifts, how many hours do you work in your regular shift? [(Both) In the last year I have worked in both 7-7-10 and 12-hour shifts]	899
4	Have you worked at CAUSA during the first waves of the COVID-19 pandemic?	0
5	Please indicate the SEVERITY of your current sleep problem(s) (e.g., within the past 2 weeks): [Difficulty falling asleep]	47
6	Please indicate the SEVERITY of your current sleep problem(s) (e.g., during the last 2 weeks): [Difficulty staying asleep]	47
7	Please indicate the SEVERITY of your current sleep problem(s) (e.g., within the last 2 weeks): [Trouble waking up too early]	47
8	How SATISFIED are you with your dream today?	47
9	To what extent do you feel your sleep problem INTERFERES with your daily functioning (e.g., fatigue during the day, ability to perform daily tasks/work, etc.)?	48
10	To what extent do you think OTHERS are aware of your sleep problem because of what it affects your quality of life?	48
11	How CONCERNED are you about your current sleep problem?	48
12	To what extent have you felt clear today?	116
13	To what extent have you felt worried today?	116
14	How frustrated have you been by the lack of sleep today?	118
15	This is an attentional question. If you are reading this, please select answer 1.	119
16	To what extent have you felt irritable today?	118

drcarlosroncero@gmail.com

17	To what extent have you felt stressed today?	118
18	To what extent have you felt energetic today?	118
19	To what extent have you felt sleepy today?	117
20	To what extent have you felt awake today?	119
21	To what extent have you felt with renewed strength today?	120
22	To what extent have you felt physical fatigue today?	121
23	To what extent have you felt mental fatigue today?	121
24	To what extent have you been able to concentrate today?	121
25	To what extent have you been forgetful today?	121
26	How much has it cost you to do your daily activities today (e.g., reading, cleaning, working, studying)?	122
27	During the last 2 weeks, how often have you felt discomfort from the following problems? [Little interest or pleasure in doing things.]	163
28	During the last 2 weeks, how often have you felt discomfort from the following problems? [Feeling discouraged, depressed, or hopeless.]	163
29	During the last 2 weeks, how often have you felt discomfort from the following problems? [Feeling nervous, anxious, or very tense.]	163
30	During the last 2 weeks, how often have you felt discomfort from the following problems? [Not being able to stop worrying or not being able to control worry.]	163
31	Over the past 4 weeks, how much has it bothered you to have trouble sleeping?	163
32	I regularly smoke more than 1 cigarette a day	167
33	A week the alcoholic beverages I drink are:	167
34	The number of coffees/teas/energy drinks I usually drink each day is: (If it's double coffee, count by 2. If it is an energy drink [330ml] count by 5).	167
35	I take sleep medication: 1 time a month or more	167
36	The medication I take to sleep is prescribed by a doctor	777
37	I take 1 time a month or more Medication for depression or anxiety	167
38	The medication I take for depression or anxiety is prescribed by a doctor	839
39	I take 1 time a month or more Illegal substances. [STIMULANTS (cocaine, amphetamines...)]	167
40	I take 1 time a month or more Illegal substances. [DEPRESSANTS (opiates or others).]	167
41	I take 1 time a month or more Illegal substances. [CANNABIS TYPE.]	167
42	I am in psychological or psychiatric treatment [Currently]	195
43	I have sequelae from having had COVID disease [Currently]	195
44	I have been diagnosed with a sleeping sickness [Currently]	195
45	I work night shifts [currently]	195
46	Due to poor sleep quality in the last month I have missed work [currently]	196
47	I have rotating shifts that cause me to frequently change my sleep hours [Currently]	195
48	I do guard duty	195
49	I have hormonal disorders related to menopause	359
50	I take a nap	195
51	In a week, the times I take a nap is:	614
52	How long does he take a nap on average?	614
53	In the course of my work, there are days of concentrated and sustained attention for long periods of time.	196
54	I take enough breaks during my workday to break the monotony and disconnect to resume adequate attention in my work.	196
55	The lack of attention in my work could directly generate serious consequences	196

drcarlosroncero@gmail.com

56	My sleep problem negatively influences my quality of life	196
57	To perform my work I need concentration and the use of higher capacities (attention, perception and memory).	196
58	I've noticed that I make more mistakes in my day-to-day tasks. [In the last month and due to poor sleep quality]	197
59	I have felt that the attention that the job demands of me is greater than what I can offer. [In the last month and due to poor sleep quality]	197
60	Perform my work [In the last month and due to poor sleep quality I have difficulty with]	197
61	Perform household chores [In the last month and due to poor sleep quality I have difficulty with]	198
62	Relate to others [In the last month and due to poor sleep quality I have difficulty with]	197
63	Have you been worried that your job was in jeopardy due to the COVID-19 pandemic?	366
64	[Phone] -Subthreshold Insomnia- You are informed that you may have problems with sleep. Occupational Medicine has set up an operation to treat specific sleep problems. In the event that there is availability...	148
65	[Email] -Subthreshold Insomnia- You are informed that you may have problems with sleep. Occupational Medicine has set up an operation to treat specific sleep problems. In the event that there is availability...	160
66	[Phone] -Clinical Insomnia- You are informed that it seems that you may have problems with sleep. Occupational Medicine has set up an operation to treat specific sleep problems. If you want to get treatment for your problem with the...	127
67	[Email] -Clinical Insomnia- You are informed that it seems that you may have problems with sleep. Occupational Medicine has set up an operation to treat specific sleep problems. If you want to get treatment for your problem with the...	133

drcarlosroncero@gmail.com
